# Three bedside techniques to quantify dynamic pulmonary hyperinflation in mechanically ventilated patients with chronic obstructive pulmonary disease

**DOI:** 10.1186/s13613-021-00948-9

**Published:** 2021-12-04

**Authors:** L. H. Roesthuis, J. G. van der Hoeven, C. Guérin, J. Doorduin, L. M. A. Heunks

**Affiliations:** 1 Department of Intensive Care Medicine, Radboud University Medical Center, Geert Grooteplein-Zuid 10, 6525 GA Nijmegen, The Netherlands; 2 Service de Medicine Intensive Réanimation, Hôpital Edouard Herriot, Lyon, France; 3 Donders Institute for Brain, Cognition and Behaviour, Department of Neurology, Radboud University Medical Center, Nijmegen, The Netherlands; 4 Department of Intensive Care Medicine, Amsterdam UMC, Location VUmc, Amsterdam, The Netherlands

**Keywords:** Chronic obstructive pulmonary disease, Dynamic pulmonary hyperinflation, Mechanical ventilation, Volume at end-inspiration, Bedside techniques

## Abstract

**Background:**

Dynamic pulmonary hyperinflation may develop in patients with chronic obstructive pulmonary disease (COPD) due to dynamic airway collapse and/or increased airway resistance, increasing the risk of volutrauma and hemodynamic compromise. The reference standard to quantify dynamic pulmonary hyperinflation is the measurement of the volume at end-inspiration (Vei). As this is cumbersome, the aim of this study was to evaluate if methods that are easier to perform at the bedside can accurately reflect Vei.

**Methods:**

Vei was assessed in COPD patients under controlled protective mechanical ventilation (7 ± mL/kg) on zero end-expiratory pressure, using three techniques in a fixed order: (1) reference standard (Vei_reference_): passive exhalation to atmosphere from end-inspiration in a calibrated glass burette; (2) ventilator maneuver (Vei_maneuver_): measuring the expired volume during a passive exhalation of 45s using the ventilator flow sensor; (3) formula (Vei_formula_): (Vt × *P*_plateau_)/(*P*_plateau_ − PEEP_i_), with Vt tidal volume, *P*_plateau_ is plateau pressure after an end-inspiratory occlusion, and PEEP_i_ is intrinsic positive end-expiratory pressure after an end-expiratory occlusion. A convenience sample of 17 patients was recruited.

**Results:**

Vei_reference_ was 1030 ± 380 mL and had no significant correlation with *P*_plateau_ (*r*^2^ = 0.06; *P* = 0.3710) or PEEP_i_ (*r*^2^ = 0.11; *P* = 0.2156), and was inversely related with *P*_drive_ (calculated as *P*_plateau_ −PEEP_i_) (*r*^2^ = 0.49; *P* = 0.0024). A low bias but rather wide limits of agreement and fairly good correlations were found when comparing Vei_maneuver_ and Vei_formula_ to Vei_reference_. Vei remained stable during the study period (low bias 15 mL with high agreement (95% limits of agreement from − 100 to 130 mL) and high correlation (*r*^2^ = 0.98; *P* < 0.0001) between both measurements of Vei_reference_).

**Conclusions:**

In patients with COPD, airway pressures are not a valid representation of Vei. The three techniques to quantify Vei show low bias, but wide limits of agreement.

**Supplementary Information:**

The online version contains supplementary material available at 10.1186/s13613-021-00948-9.

## Introduction

**Fig. 1:**
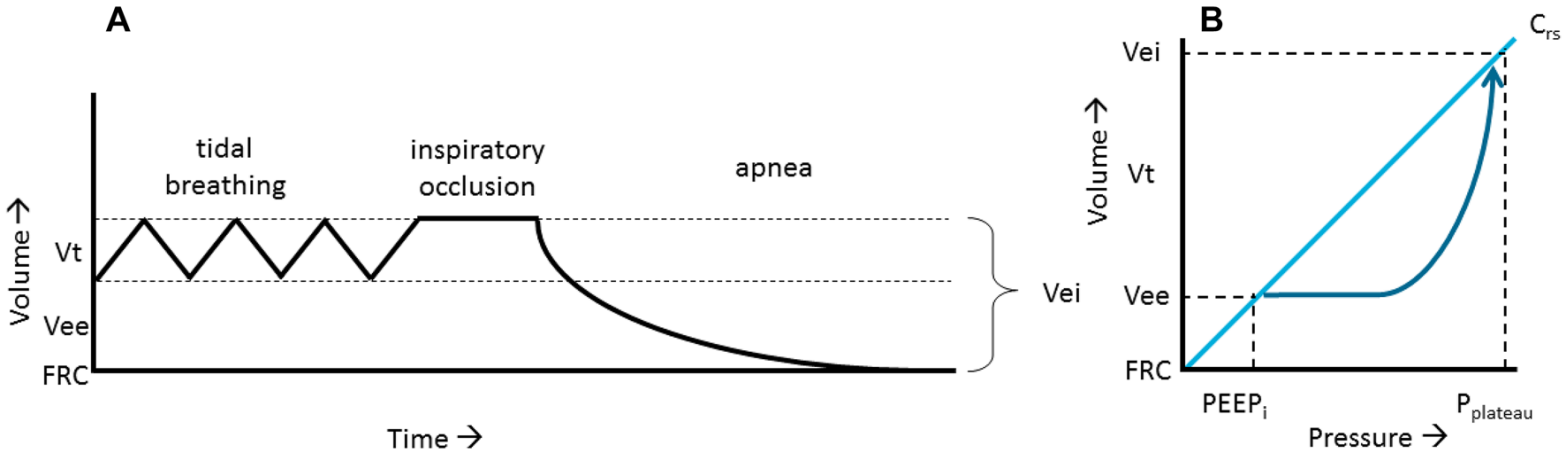
**A** Schematic representation of the volume at end-inspiration (Vei), which is the volume at end-expiration (Vee) above the functional residual capacity plus tidal volume, measured after prolonged apnea. **B** Schematic representation explaining the rationale of the formula to estimate Vei, with pressure on the x-axis and volume on the y-axis. In a patient with dynamic pulmonary hyperinflation inspiration starts from the total amount of positive end-expiratory pressure (PEEP_total_). PEEP_total_ can be obtained by performing an end-expiratory occlusion maneuver (i.e., zero flow conditions, after an occlusion of a few seconds PEEP_total_ represents the alveolar pressure). If applied PEEP by the ventilator is 0 cmH_2_O, which is the case in the current study, PEEP_total_ represents intrinsic PEEP (PEEP_i_). The patient inhales a certain volume (Vt) reaching an inspiratory pressure depending on the mechanical characteristics of the lung. By performing an end-inspiratory occlusion maneuver the plateau pressure (*P*_plateau_) can be obtained, which corresponds to Vei. Compliance (*C*_rs_) is defined as the slope of the volume − pressure relationship, e.g., the ratio of a change in volume and pressure, for the respiratory system this means: Vt/(*P*_plateau_ −PEEP_i_) (1). From the figure it is clear that *C*_rs_ can also be calculated as Vei/*P*_plateau_ (2). Therefore, Vei is Vee plus Vt, but also C_rs_ times *P*_plateau_ (3). Combining [1], (2) and (3) gives Vei_formula_ = (Vt)/*P*_plateau_–PEEP_i_) * *P*_plateau_ (4) which can be rewritten as Vei_formula_ = (Vt * *P*_plateau_)/(*P*_plateau_ –PEEP_i_) (5). This rationale holds true when *C*_rs_ remains constant

Dynamic pulmonary hyperinflation is defined as increased relaxation volume of the respiratory system at the end of a tidal expiration above the expected normal value [1]. Dynamic pulmonary hyperinflation is a cardinal feature in patients with chronic obstructive pulmonary disease (COPD) and results from dynamic airway collapse and/or increased airway resistance. Furthermore, as highlighted by the word *dynamic*, dynamic pulmonary hyperinflation is a consequence of a discrepancy between the expiratory time constant and the expiratory time either adopted by the patient or set at the ventilator [1]. It increases the risk of volutrauma and hemodynamic compromise, especially during invasive mechanical ventilation and therefore should be monitored in both the intensive care unit (ICU) and operation theater [2]. The presence of dynamic pulmonary hyperinflation should be considered if expiratory flow does not cease at the end of expiration [1], although expiratory flow may be close to zero in severe expiratory flow limitation [2]. The reference technique to quantify dynamic pulmonary hyperinflation is measurement of the volume at end-inspiration (Vei) [3]. As shown in Fig. 1, Vei is the volume of air exhaled passively from end-inspiration to end-expiration at functional residual capacity (FRC). Williams and Tuxen showed almost 30 years ago that Vei best predicted the risk of volutrauma (pneumothorax and/or subcutaneous emphysema) and hypotension, and suggested to maintain Vei below 1400 mL (or 20 mL/kg predicted body weight) [4, 5]. Measurement of Vei requires specific equipment and is cumbersome, therefore intrinsic positive end-expiratory pressure (PEEP_i_) or end-inspiratory plateau pressure (*P*_plateau_) is commonly used as surrogates to quantify hyperinflation [6, 7]. Often, PEEP_i_ is in the range of 10–15 cmH_2_O in patients with severe airway obstruction [8]. Maintaining *P*_plateau_ below 25–30 cmH_2_O is mostly suggested to prevent risks of volutrauma and hypotension [6]. Safe upper limits for *P*_plateau_ and PEEP_i_ to limit risk of complications, however, are not well defined [6]. As airway pressures (PEEP_i_, *P*_plateau_) at a certain lung volume (e.g., Vei) depend on respiratory elastance, safe airway pressures do not necessarily reflect safe Vei.

The aims of the current study were to evaluate in patients with severe COPD under controlled mechanical ventilation: (1) if airway pressures (i.e., *P*_plateau_ and PEEP_i_) are valid representations of Vei, and (2) if two methods to quantify Vei, which are easier to perform at the bedside, namely a simple physiology-based equation and the use of ventilator built-in equipment to measure Vei, could provide a valid alternative to its direct measurement.

## Methods

### Study design and population

This is an observational study in patients admitted to the Intensive Care Unit of the Radboud University Medical Center (Nijmegen, the Netherlands). Inclusion criteria were acute exacerbation of COPD, volume controlled mechanical ventilation, deep sedation and neuromuscular blockade. Patients with FiO_2_ > 0.70 or volutrauma (pneumothorax or pneumomediastinum/subcutaneous emphysema) were excluded. The protocol was approved by the local ethical committee. Written informed consent was obtained from the legal representative before inclusion.

### Trial design and data acquisition

Patients were studied in supine position with the head of the bed elevated 30° from horizontal position. They were ventilated with the Servo-i ventilator (Maquet, Sweden) using Fisher and Paykel (Auckland, New Zealand) breathing circuit (RT380) and heated humidifier (MR850). The compensation algorithm for circuit compliance was checked before use and running in all patients. After enrollment, arterial blood from an indwelling catheter was withdrawn and ventilator settings remained as set by treating clinicians. Vei was measured using three different techniques, which were applied in the following non-randomized order:

1. Reference standard (Vei_reference_): during an end-inspiratory occlusion the endotracheal tube was briefly occluded with dedicated Kocher scissors (Additional file 1: Figure S1). The patient was disconnected from the ventilator and the endotracheal tube was immediately connected to a calibrated glass burette with a soap film bubble [9]. After release of the Kocher scissors, passive expiration was allowed and the exhaled volume was measured (Additional file 1: Figure S2). The setup was calibrated using a 500-mL calibration syringe. The exhaled volume was corrected for body temperature (BTPS correction factor 1.091 at ambient temperature 22 °C).
2. Ventilator maneuver (Vei_maneuver_): while in volume controlled mode, an end-inspiratory occlusion was performed. While holding the ventilator knob for the occlusion maneuver, the ventilator was switched to pressure support mode with back-up time of 45 seconds, after which the end-inspiratory occlusion knob was released. Expiratory flow was measured with the built-in ventilator flow sensor (measurement range 0–3.2 L/s and inaccuracy of expiratory volume between 100 and 4000 mL is ± 4 mL + 8% of true volume, and between 2 and 100 mL is ± 2.5 mL + 10% of true volume (Maquet, Sweden). Vei_maneuver_ was defined as the expired volume after a 45-s passive exhalation, which could be obtained from the ventilator screen.
3. Formula (Vei_formula_): the formula is deciphered as follows (Fig. 1):
  1 $$C_{{{\text{rs}}}} = \, {{{\text{Vt}}} \mathord{\left/ {\vphantom {{{\text{Vt}}} {\left( {P_{{{\text{plateau}}}} {-}{\text{ PEEP}}_{{\text{i}}} } \right)}}} \right. \kern-\nulldelimiterspace} {\left( {P_{{{\text{plateau}}}} {-}{\text{ PEEP}}_{{\text{i}}} } \right)}},$$
  with Vt the tidal volume and *C*_rs_ the static compliance of the respiratory system. *P*_plateau_ and PEEP_i_ were measured 3–5 seconds after an end-inspiratory and end-expiratory occlusion, respectively, ensuring stable plateau pressures (i.e., no external PEEP was applied, therefore total amount of PEEP was equal to PEEPi).
  2 $$C_{{{\text{rs}}}} = \, {{{\text{Vei}}_{{{\text{formula}}}} } \mathord{\left/ {\vphantom {{{\text{Vei}}_{{{\text{formula}}}} } {P_{{{\text{plateau}}}} }}} \right. \kern-\nulldelimiterspace} {P_{{{\text{plateau}}}} }}$$
  3 $${\text{Vei}}_{{{\text{formula}}}} = \, C_{{{\text{rs}}}} * \, P_{{{\text{plateau}}}}$$
  4 $${\text{Vei}}_{{{\text{formula}}}} = {{{\text{Vt}}} \mathord{\left/ {\vphantom {{{\text{Vt}}} {\left( {P_{{{\text{plateau}}}} {-}{\text{ PEEPi}}} \right)}}} \right. \kern-\nulldelimiterspace} {\left( {P_{{{\text{plateau}}}} {-}{\text{ PEEPi}}} \right)}}*P_{{{\text{plateau}}}}$$
  5 $${\text{Vei}}_{{{\text{formula}}}} = {{\left( {{\text{Vt }}* \, P_{{{\text{plateau}}}} } \right)} \mathord{\left/ {\vphantom {{\left( {{\text{Vt }}* \, P_{{{\text{plateau}}}} } \right)} {\left( {P_{{{\text{plateau}}}} {-}{\text{ PEEP}}_{{\text{i}}} } \right)}}} \right. \kern-\nulldelimiterspace} {\left( {P_{{{\text{plateau}}}} {-}{\text{ PEEP}}_{{\text{i}}} } \right)}}$$
  It is assumed that *C*_rs_ remains constant during expiration.

Vei was measured using these three techniques in each patient with at least 5-min interval between measurements. Before prolonged passive expiration (Vei_reference_ and Vei_maneuver_) patients were preoxygenated with FiO_2_ 1.0 for 1 min. Peripheral oxygen saturation, heart rate and arterial blood pressure were continuously monitored. To confirm that no changes in Vei developed during the study protocol and to test repeatability, Vei_reference_ was repeated after the three techniques and compared to the initial Vei_reference_.

### Statistical analysis

A data analysis and a statistical plan were written after the data were accessed. Statistical analysis was performed with Prism 5 (GraphPad Software, San Diego, CA, USA). Continuous data were tested for normality using the Shapiro–Wilk test and presented as mean ± standard deviation (SD) and range. Linear regression analysis was used to model the relationships between airway pressures and Vei_reference_, and to compare Vei_maneuver_ and Vei_formula_ with Vei_reference_. The relationship of difference to average between the variables was tested using the Bland–Altman representation that provided bias and 95% limits of agreement. Furthermore, linear regression analysis was performed of the difference on the average. A two-tailed *P* < 0.05 was considered statistically significant. Being a physiological study, a convenience sample of 17 patients was recruited and considered appropriate.

## Results

Data at study enrollment are shown in Table 1. Patients had severe airway obstruction, indicated by normal to high *C*_rs_ (58 ± 19 (range 27–96) mL/cmH_2_O), high resistance of the respiratory system (27 ± 9 (range 16–50) cmH_2_O/L/s), resulting in a long time constant (1.5 ± 0.6 (range 0.6–2.7) s). Endotracheal tube diameter of the patients was 7.5 ± 0.5 mm. In one patient it was not feasible to measure Vei_reference_ due to technical failure of the reference technique, therefore 16 patients were analyzed. No adverse events were reported during the study.

**Table 1:** Baseline parameters

Gender (M/F)	7/9
Age (yr)	63 ± 10
Height (m)	1.70 ± 0.10
Actual body weight (kg)	75 ± 15
Body mass index (kg/m^2^)	25.9 ± 4.5
Days of mechanical ventilation	2.2 ± 1.6
Blood pressure (S/D, mmHg)	122 ± 17 / 59 ± 6
Ventilatory settings	
RR (breaths/min)	15 ± 5
Vt (mL)	438 ± 47
Vt/PBW (mL/kg)	7 ± 1
Ti (s)	0.7 ± 0.2
Te (s)	3.6 ± 1.2
Respiratory mechanics	
P_peak_ (cmH_2_O)	35 ± 7 (range 19–47)
* P*_plateau_ (cmH_2_O)	18 ± 4 (range 10–24)
PEEP_i_ (cmH_2_O)	9 ± 3 (range 3–14)
R_rs_ (cmH_2_O/L/s)	27 ± 9 (range 16–50)
* C*_rs_ (mL/cmH_2_O)	58 ± 19 (range 27–96)
Time constant (s)	1.5 ± 0.6 (range 0.6–2.7)
Arterial blood gas	
pH	7.30 ± 0.07
PaO_2_ (mmHg)	93 ± 25
PaCO_2_ (mmHg)	65 ± 16
HCO_3_^−^ (mmol/L)	31 ± 6

Patients were ventilated with zero applied positive end-expiratory pressure. Data are presented as mean ± SD and range (if mentioned)

*S/D*, systolic/diastolic; RR, respiratory rate; Vt, tidal volume; Vt/PBW, tidal volume normalized for predicted body weight; Ti, inspiratory time; Te, expiratory time; *P*_peak_, peak pressure; *P*_plateau_, plateau pressure; PEEP_i_, intrinsic positive end-expiratory pressure; *R*_rs_, resistance of respiratory system; *C*_rs_, compliance of respiratory system, HCO_3_^−^ plasma bicarbonate value

### Correlation between Vei and airway pressures

**Fig. 2:**
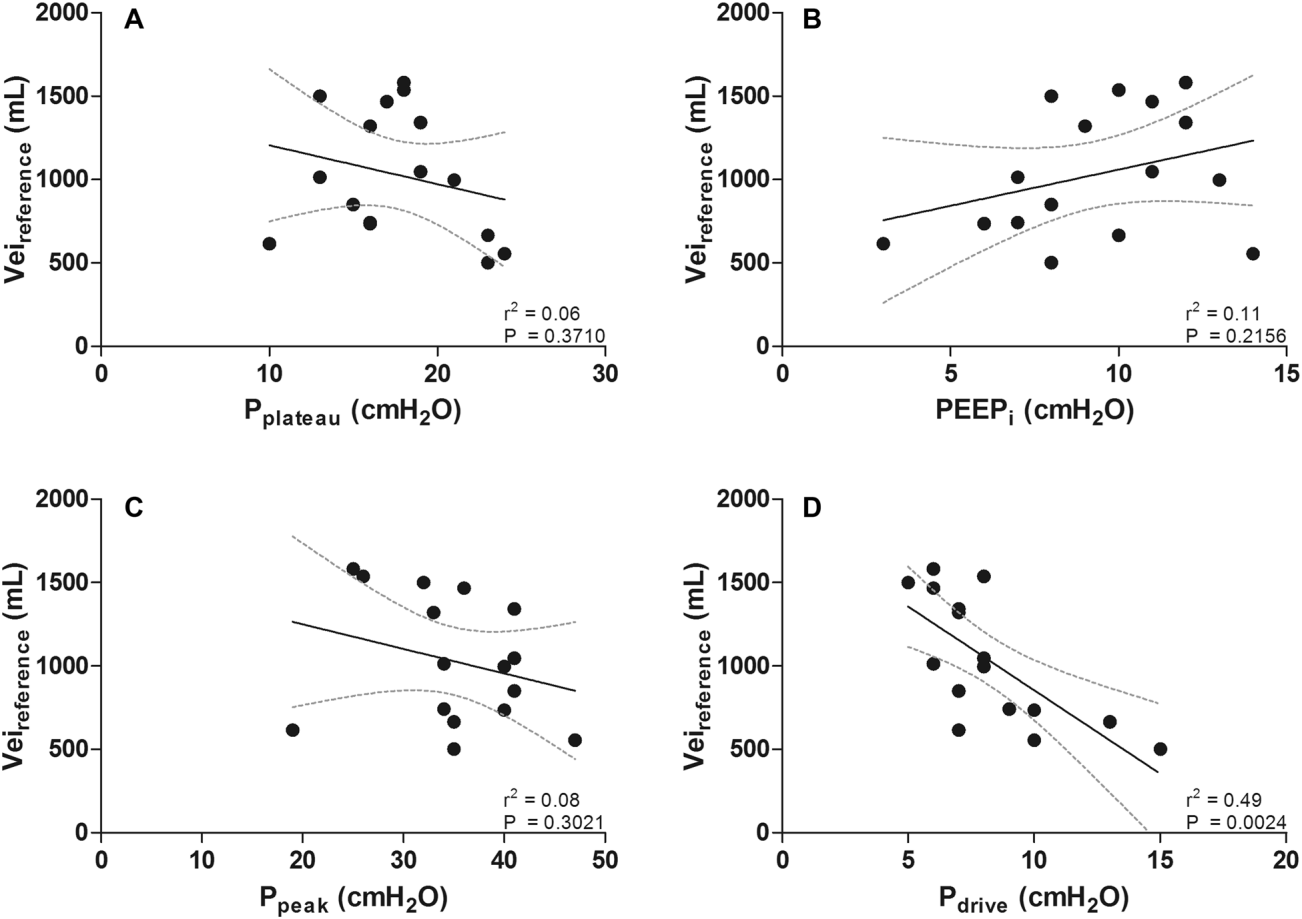
Airway pressures are commonly used to quantify hyperinflation, especially *P*_plateau_ and PEEP_i_. No correlation was found between *P*_plateau_ and Vei_reference_ (**A**) nor between PEEP_i_ and Vei_reference_ (**B**) or between P_peak_ and Vei_reference_ (**C**) (solid line with dashed 95% confidence interval (CI) lines). The driving pressure (*P*_drive_) was significantly correlated with Vei_reference_ (**D**)

Figure 2 shows no significant correlation between *P*_plateau_ and Vei_reference_ (*r*^2^ = 0.06; *P* = 0.3710), between PEEP_i_ and Vei_reference_ (*r*^2^ = 0.11; *P* = 0.2156) and between *P*_peak_ and Vei_reference_ (*r*^2^ = 0.08; *P* = 0.3021). The driving pressure (*P*_drive_, calculated as *P*_plateau_ − PEEP_i_) was inversely related with Vei_reference_ (*r*^2^ = 0.49; *P* = 0.0024), consequently a moderate positive correlation with almost the same correlation coefficient was found between *C*_rs_ and Vei_reference_ (*r*^2^ = 0.50; *P* = 0.0023) (Additional file 1: Table S1). The correlations between airway pressures and Vei_reference_ corrected for predicted body weight are reported in Additional file 1: Table S1.

**Fig. 3:**
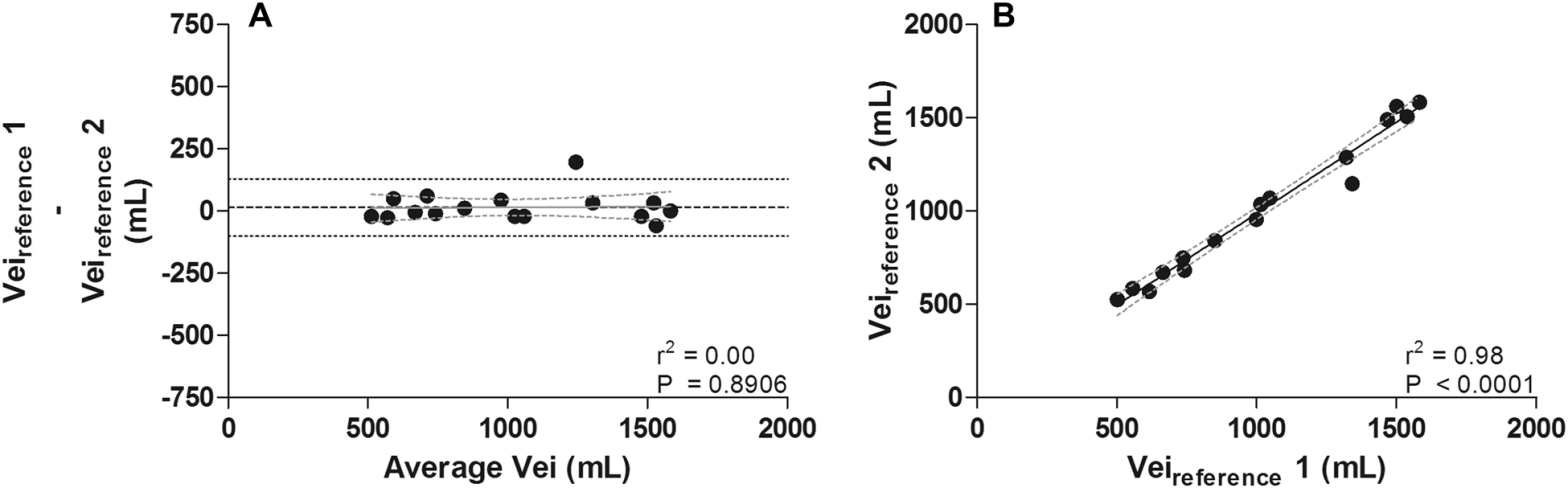
Dynamic pulmonary hyperinflation did not change during the study protocol: there was a low bias with high agreement (**A**) and high correlation between the two measurements of Vei_reference_ (**B**) (solid line with dashed 95% CI lines)

Vei_reference_ did not change during the study and measurement according to the reference standard had a high repeatability: a low bias of 15 mL with high agreement (from − 100 to 130 mL) and high correlation (*r*^2^ = 0.98; *P* < 0.0001) were found between the first and last measurement of Vei_reference_ (Fig. 3).

### Comparison of three bedside techniques to quantify hyperinflation

**Fig. 4:**
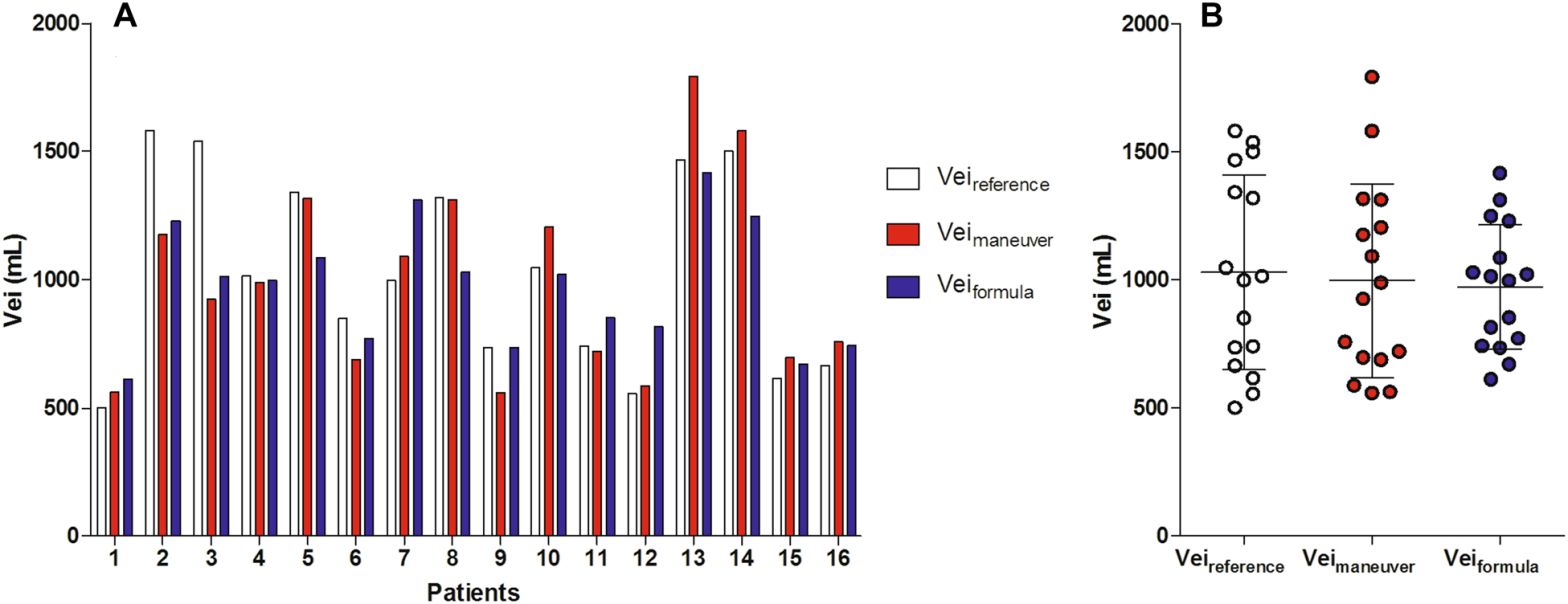
Individual (**A**) and mean ± SD (**B**) data of the different bedside techniques to quantify dynamic pulmonary hyperinflation

**Fig. 5:**
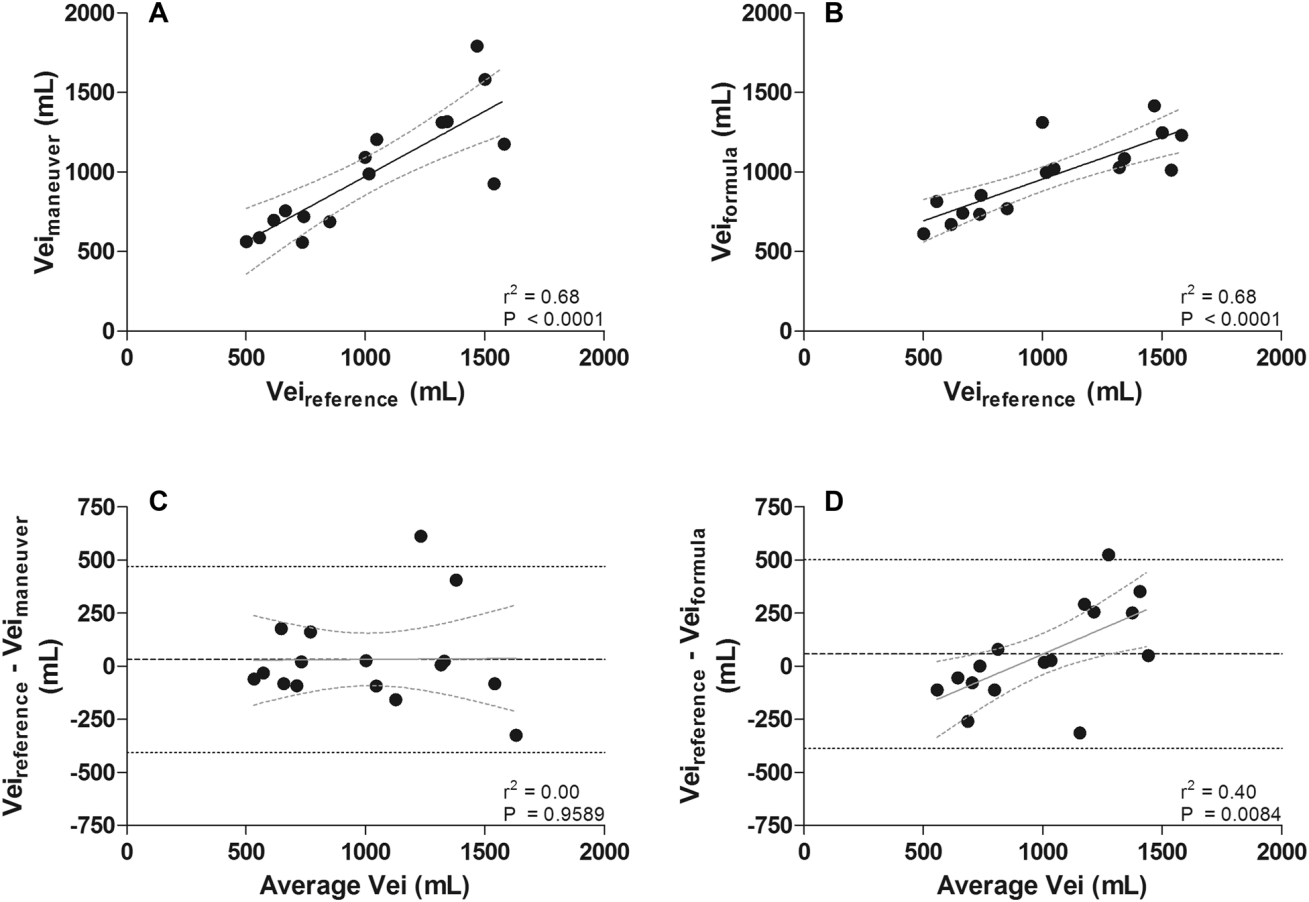
Bedside techniques to quantify dynamic pulmonary hyperinflation compared with the gold standard (Vei_reference_). Fairly good correlations were found between Vei_reference_ and Vei_maneuver_ (**A**) and between Vei_reference_ and Vei_formula_ (**B**). Bland–Altman analysis showed low bias and wide limits of agreement between Vei_reference_ and Vei_maneuver_ (**C**) and between Vei_reference_ and Vei_formula_ (**D**). Furthermore, there is a relationship in the bias between Vei_reference_ and Vei_formula_ (solid line with dashed 95% CI lines)

Vei_reference_ was 1030 ± 380 mL, Vei_maneuver_ was 998 ± 377 mL and Vei_formula_ was 972 ± 243 mL (Fig. 4). Fairly good correlations were found when comparing Vei_maneuver_ and Vei_formula_ to Vei_reference_ (Fig. 5A, B). A low bias but relatively wide limits of agreement were found when comparing Vei_maneuver_ (bias 32 mL, from − 406 to 470 mL) and Vei_formula_ (bias 58 mL, from − 387 to 502 mL) to Vei_reference_ (Fig. 5C, D), a significant relationship in bias was found for the latter (*r*^2^ = 0.40; *P* = 0.0084), suggesting that bias is related to the magnitude of measurements. The comparisons and correlations for Vei_maneuver_ and Vei_formula_ with Vei_reference_ corrected for predicted body weight are shown in Additional file 1: Figure S3. *C*_rs_ was moderately correlated with Vei_maneuver_ (*r*^2^ = 0.60; *P* = 0.0004) and Vei_formula_ (*r*^2^ = 0.53; *P* = 0.0013) (Additional file 1: Table S1).

## Discussion

The main findings of our study can be summarized as follows: in invasively ventilated patients with acute exacerbation of COPD (1) Vei_reference_ is not significantly correlated with PEEP_i_, *P*_plateau_ or *P*_peak_. Vei_reference_ is inversely correlated with *P*_drive_; (2) Vei_reference_ is significantly correlated with Vei measured with the ventilator maneuver or when calculated using a physiology-based formula and has a low bias, but rather wide limits of agreement. When accepted that Vei_reference_ is the reference standard to quantify pulmonary hyperinflation, the current study suggests that both airway pressures (*P*_plateau_ and PEEP_i_) and the two alternative methods to measure Vei, perform only moderately in clinical practice.

### Airway pressures to estimate Vei

In clinical practice, PEEP_i_ or *P*_plateau_ are measured to estimate alveolar pressures in patients with COPD. The recommended safe upper limit for *P*_plateau_ is below 25–30 cmH_2_O [6, 7]. However, in our study no significant correlation was found between *P*_plateau_ and Vei or PEEP_i_ and Vei. In fact, *P*_plateau_ was lower than 20 cmH_2_O in four patients, despite a Vei > 1400 mL. Williams [5] reported that complications due to pulmonary hyperinflation developed only when Vei was > 1400 mL, although it should be mentioned that the study sample was too small to provide strong clinical recommendations. It should be recognized that previous studies [4, 5] also recruited patients with asthma, while we included only COPD patients. Previously, a poor correlation was reported between *P*_plateau_ and Vei, but in that study [5] no end-inspiratory occlusion (i.e., only a pause time of 0.5 s was applied) was performed and PEEP_i_ was calculated instead of being measured. When accepting that complications (pneumothorax, subcutaneous emphysema) related to mechanical ventilation in patients with obstructive airway disease result from increased end-expiratory lung volume, assessment of volume (e.g., Vei) seems preferable. In this case, airway pressure, and therefore *P*_plateau_ and PEEP_i_ should be used cautiously to monitor dynamic pulmonary hyperinflation. The lack of correlation between *P*_plateau_ and Vei_reference_ or PEEP_i_ and Vei_reference_ (even when corrected for predicted body weight), is somewhat surprising. On the other hand, hemodynamic compromise in these patients is associated with increased intrathoracic pressure and may be better monitored with airway pressure (*P*_plateau_, PEEP_i_). Obviously, volume and pressure in the respiratory system are coupled by respiratory elastance (inverse of compliance). The absence of a significant correlation between pressures and volumes (both Vei and Vt) indicate non-linearity of the respiratory elastance over the range of volumes used in measurements. We found an inverse relationship between *P*_drive_ and Vei_reference_ (absolute value or corrected for predicted bodyweight): patients with low *P*_drive_ (calculated as *P*_plateau_ −PEEP_i_) have higher Vei, and vice versa. Possibly, patients with higher *inspiratory* driving pressure, may also have higher *expiratory* driving pressure (pressure difference between alveoli and airway opening during expiration) and as such limit pulmonary hyperinflation. Alternatively, an *inspiratory* lower driving pressure indicates higher compliance of the respiratory system (at constant Vt), causing a reduction in expiratory flow due to lower elastic recoil pressure, thereby promoting hyperinflation. This should be verified in future studies using esophageal and gastric balloons.

### Different methods to quantify Vei

We compared two techniques to quantify Vei against the reference method. Although Vei measured with the ventilator maneuver is easy to perform at the bedside, without the need to disconnect the patients from the ventilator circuit, a limitation is that this maneuver cannot be performed with every type of ventilator (it requires change of mode, with persistent end-inspiratory occlusion). The variables required to calculate Vei with the formula can be obtained with every modern ICU ventilator and does not require disconnection of the patient from the ventilator.

Despite significant correlations and low bias between Vei_reference_ and the other two techniques, we found rather wide limits of agreement between Vei_reference_ and the two other bedside techniques to quantify Vei. Vei_reference_ was similar at the start and end of the study protocol, virtually excluding biological variation as an explanation for these wide limits of agreement. A possible explanation for the suboptimal performance of Vei_maneuver_ compared to Vei_reference_ is that expired volume measured by the ventilator may deviate from the actual tidal volume due to application of ventilator algorithms that compensate for gas compression and changes in humidification/temperature. For the Servo-i ventilator used in this study, this means that delivered tidal volume can be 15–25% higher compared to set tidal volume [10]. The moderate performance of Vei_formula_ may be related to the assumption that compliance remains the same over a wide volume range (Fig. 1). Small airway closure makes the relationship between volume and pressure nonlinear below end-expiratory lung volume [11]. Another important factor possibly explaining the wide limits of agreement between Vei_reference_ and the other two bedside methods to quantify Vei, is expiratory valve resistance. When measuring Vei_reference_, patients exhaled to atmosphere, bypassing the expiratory valve of the ventilator, but this is not true for the other two methods. Recently, Pinède et al. [12] performed a bench study and found that expiratory valve resistance highly differed among the ventilators that were tested. For the Servo-u (the Servo-i was not tested) expiratory valve resistance increased when higher PEEP levels were applied, increasing from 13.8 cmH_2_O/L/s at PEEP 5 cmH_2_O to 39.5 cmH_2_O/L/s at PEEP 15 cmH_2_O. The same might be true for PEEP_i_, resulting in a PEEP_i_-depending difference between Vei_reference_ and the other two methods.

The present study shows that when Vei_reference_ is accepted as the reference technique, both static airway pressures (*P*_plateau_ and PEEP_i_) and Vei obtained from alternative techniques should be interpreted cautiously. In addition, the safe limits for Vei_reference_ are derived from small studies [4, 5]. Despite the fact that Vei_reference_ is not widely adopted in clinical care, the incidence of clinical complications due to pulmonary hyperinflation has decreased since the initial studies describing development of pulmonary hyperinflation (for review [1]). Apparently, more widespread use of lung protective ventilation (limiting minute ventilation and Vt) and permissive hypercapnia were already successful in decreasing the incidence of complications due to pulmonary hyperinflation. Our formula for Vei is easy to use at the bedside and may be a reasonable alternative to quantify hyperinflation when the chest wall has a disproportional impact on *P*_plateau_. However, use of Vei_formula_ carries the risk of underestimating dynamic pulmonary hyperinflation at relatively high levels of Vei.

### Strengths and limitations

The strengths of this study include novelty of the data. It is surprising that 30 years after the classical studies by Tuxen and colleagues [3–5, 13], Vei has not been compared to *P*_plateau_ and PEEP_i_, parameters commonly used in clinical practice to quantify dynamic hyperinflation. Also, although bedside tests to estimate Vei are much needed, this study shows that available techniques do not perform adequately. The data from this study are of clinical importance and also of value for future trials that aim to test the effects of interventions on dynamic hyperinflation in patients with COPD. Also, we used a reference standard to assess Vei [9]. In the original studies by Tuxen [3–5, 13], Vei was measured using 2 calibrated 2.2-L volumetric spirometers (Puritan Bennett), although it is unclear if these were directly connected to the endotracheal tube, or in the ventilator circuit. Care should be taken when measuring Vei from the ventilator circuit, given the presence of bias flow delivered by the ventilator. Another strength of the study is the high repeatability of Vei_reference_, therefore poor agreement with the other methods cannot be explained by poor repeatability of the reference standard [14].

Several limitations should be addressed. First, this is a single-center physiological study without sample size analysis and patients were recruited only when the study team was available. Second, we did not randomize the order of the techniques to measure Vei. However, the high repeatability and low bias in the Bland–Altman plots of Vei_reference_ (measured at beginning and end of the study), makes it extremely unlikely that Vei changed during the course of the study. Third, patients had a severe airway obstruction, as indicated by a long time constant, but *P*_plateau_ was below 25 cmH_2_O in all patients and Vei measured with the reference standard above 1400 mL in only four patients. This may due to the fact that data were obtained in an ICU with expertise in mechanical ventilation and quantification of Vei using Vei_formula_ is part of the clinical protocol. Indeed, none of the patients developed complications associated with dynamic pulmonary hyperinflation. Also, it should be noted that there is increased attention for lung protective ventilation in the last decade as compared to 30 years ago, thereby limiting minute ventilation and especially tidal volumes (i.e., both were almost halved in our study as compared to Williams et al. [5]). However, it should be acknowledged that severe complications resulting from dynamic pulmonary hyperinflation are still reported, highlighting the need for bedside monitoring tools to quantify dynamic pulmonary hyperinflation [1, 6, 7]. Fourth, we did not change ventilator settings. Fifth, we did not measure airway opening pressure, which may exist above PEEP_i_ and, if present, affects calculations of respiratory mechanics [15]. However, it is not expected that this would lead to differences between the methods. Sixth, expiratory flow limitation is an important contributing factor of dynamic pulmonary hyperinflation [16–18], which has not been assessed in this study. If expiratory flow limitation is present in a patient, one would expect that it would disappear with a long expiratory time, which is the case using Vei_reference_ and Vei_maneuver_. Alveoli with the longest time constants (i.e., with the highest PEEP_i_) may remain closed during an occlusion maneuver [1] and therefore lead to underestimation of Vei using the formula. Seventh, measurements are only feasible under fully controlled mechanical ventilation. Finally, there is no firm threshold for a safe Vei.

## Conclusions

Hemodynamic consequences and increased risk of volutrauma resulting from severe dynamic pulmonary hyperinflation in patients with COPD and asthma has been reported 3 decades ago. However, quantification of dynamic pulmonary hyperinflation is seldom performed at the bedside as state of the art techniques are cumbersome. In the current study, bedside techniques to quantify dynamic pulmonary hyperinflation were evaluated in patients with severe COPD. We conclude that end-inspiratory and end-expiratory occlusion pressures are not valid representations of Vei_reference_. A physiology-based formula to estimate Vei_reference_ shows excellent correlation and low bias, but the wide limits of agreement should be recognized.

## Supplementary Information

**Additional file 1.** Additional figures and table.

## Data Availability

The datasets used and analyzed during the current study are available from the corresponding author on reasonable request.

## Abbreviations

BTPS: Body temperature pressure saturated
CI: Confidence interval
COPD: Chronic obstructive pulmonary disease
*C*_rs_: Compliance of the respiratory system
ICU: Intensive Care Unit
PBW: Predicted body weight
*P*_drive_: Driving pressure of the respiratory system
PEEP_i_: Intrinsic positive end-expiratory pressure
PEEP_total_: Total amount of positive end-expiratory pressure
*P*_peak_: Peak airway pressure of the respiratory system
*P*_plateau_: Plateau pressure of the respiratory system
RR: Respiratory rate
*R*_rs_: Resistance of the respiratory system
S/D: Systolic/diastolic
SD: Standard deviation
TC: Time constant
Ti: Inspiratory time
Te: Expiratory time
Vei: End-inspiratory lung volume
Vee: End-expiratory lung volume
Vt: Tidal volume
