# Supplementary material for: Three bedside techniques to quantify dynamic pulmonary hyperinflation in mechanically ventilated patients with chronic obstructive pulmonary disease
Source: Ann Intensive Care. 2021 Dec 4;11:167. doi: 10.1186/s13613-021-00948-9 (PMC8643378; doi:10.1186/s13613-021-00948-9)
Supplement: Supplementary file 1 — Additional file 1. Additional figures and table. [file 13613_2021_948_MOESM1_ESM.docx]

**Three bedside techniques to quantify dynamic pulmonary hyperinflation in mechanically ventilated patients with chronic obstructive pulmonary disease**

**Online Supplement**

L.H. Roesthuis M.Sc.^1^; J.G. van der Hoeven M.D., Ph.D.^1^; C. Guérin, M.D., Ph.D.^2^; J. Doorduin Ph.D.^3^; L.M.A. Heunks M.D., Ph.D.^4^

^1^ Department of Intensive Care Medicine, Radboud University Medical Center, Nijmegen, The Netherlands; ^2^ Service de Medicine Intensive Réanimation, Hôpital Edouard Herriot, Lyon, France; ^3^ Donders Institute for Brain, Cognition and Behaviour, Department of Neurology, Radboud University Medical Center, Nijmegen, The Netherlands; ^4^ Department of Intensive Care Medicine, Amsterdam UMC, location VUmc, The Netherlands

**FIGURE LEGENDS**

**Figure E1** Photo showing dedicated Kocher scissors.

**Figure E2** Photo showing the calibrated glass burette to measure expired volume.

**Figure E3** Bedside techniques to quantify dynamic pulmonary hyperinflation compared with the gold standard (Vei_reference_), with Vei corrected for predicted body weight. Bland-Altman analysis showed comparable results when Vei was not corrected for predicted body weight: a low bias and wide limits of agreement between Vei_reference_ and Vei_maneuver_ (**A**) and between Vei_reference_ and Vei_formula_ (**B**). Furthermore, there is a relationship in the bias between Vei_reference_ and Vei_formula_ (solid line with dashed 95% confidence (CI) lines). In contrast, only moderate correlations were found between Vei_reference_ and Vei_maneuver_ (**C**) and between Vei_reference_ and Vei_formula_ (**D**) when Vei was corrected for predicted body weight.

**TABLES**

**Table E1 Correlations between Vei methods and respiratory mechanics**

| **Parameter** |  | **P_plateau_** | **PEEP_i_** | **P_drive_** | **R_rs_** | **C_rs_** | **TC** |
| --- | --- | --- | --- | --- | --- | --- | --- |
| Vei_reference_ | total | r^2^ = 0.06  P = 0.3710 | r^2^ = 0.11  P = 0.2156 | r^2^ = 0.49  P = 0.0024 | r^2^ = 0.08  P = 0.2868 | r^2^ = 0.50  P = 0.0023 | r^2^ = 0.12  P = 0.1908 |
|  | Corrected PBW | r^2^ = 0.02  P = 0.6452 | r^2^ = 0.17  P = 0.1088 | r^2^ = 0.40  P = 0.0089 | r^2^ = 0.01  P = 0.7235 | r^2^ = 0.26  P = 0.0460 | r^2^ = 0.10  P = 0.2330 |
| Vei_maneuver_ | total | r^2^ = 0.04  P = 0.4434 | r^2^ = 0.11  P = 0.2117 | r^2^ = 0.43  P = 0.0057 | r^2^ = 0.00  P = 0.9696 | r^2^ = 0.60  P = 0.0004 | r^2^ = 0.42  P = 0.0068 |
|  | Corrected PBW | r^2^ = 0.00  P = 0.8375 | r^2^ = 0.18  P = 0.0988 | r^2^ = 0.29  P = 0.0306 | r^2^ = 0.07  P = 0.3152 | r^2^ = 0.31  P = 0.0259 | r^2^ = 0.47  P = 0.0035 |
| Vei_formula_ | total | r^2^ = 0.01  P = 0.7895 | r^2^ = 0.28  P = 0.0369 | r^2^ = 0.45  P = 0.0045 | r^2^ = 0.00  P = 0.8813 | r^2^ = 0.53  P = 0.0013 | r^2^ = 0.31  P = 0.0260 |
|  | Corrected PBW | r^2^ = 0.05  P = 0.4165 | r^2^ = 0.47  P = 0.0035 | r^2^ = 0.17  P = 0.1104 | r^2^ = 0.15  P = 0.1330 | r^2^ = 0.08  P = 0.2808 | r^2^ = 0.25  P = 0.0505 |

*Definition of abbreviations:* P_plateau_ = plateau pressure; PEEP_i_ = intrinsic positive end-expiratory pressure; R_rs_ = resistance of respiratory system; C_rs_ = compliance of respiratory system; TC = time constant; Vei = volume at end-inspiration; PBW = predicted body weight

**FIGURES**

**Figure E1**

**
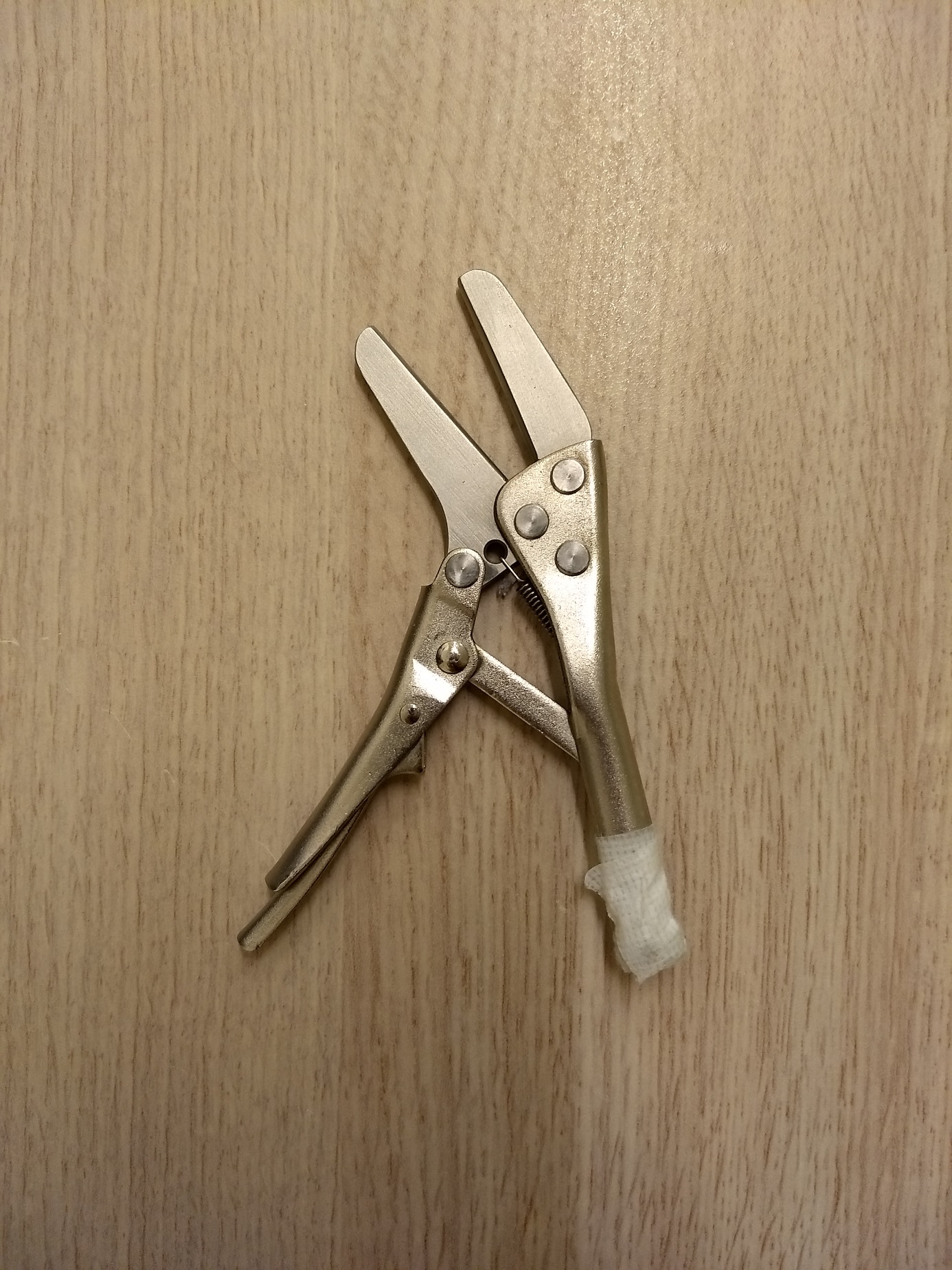
**

**Figure E2**

**
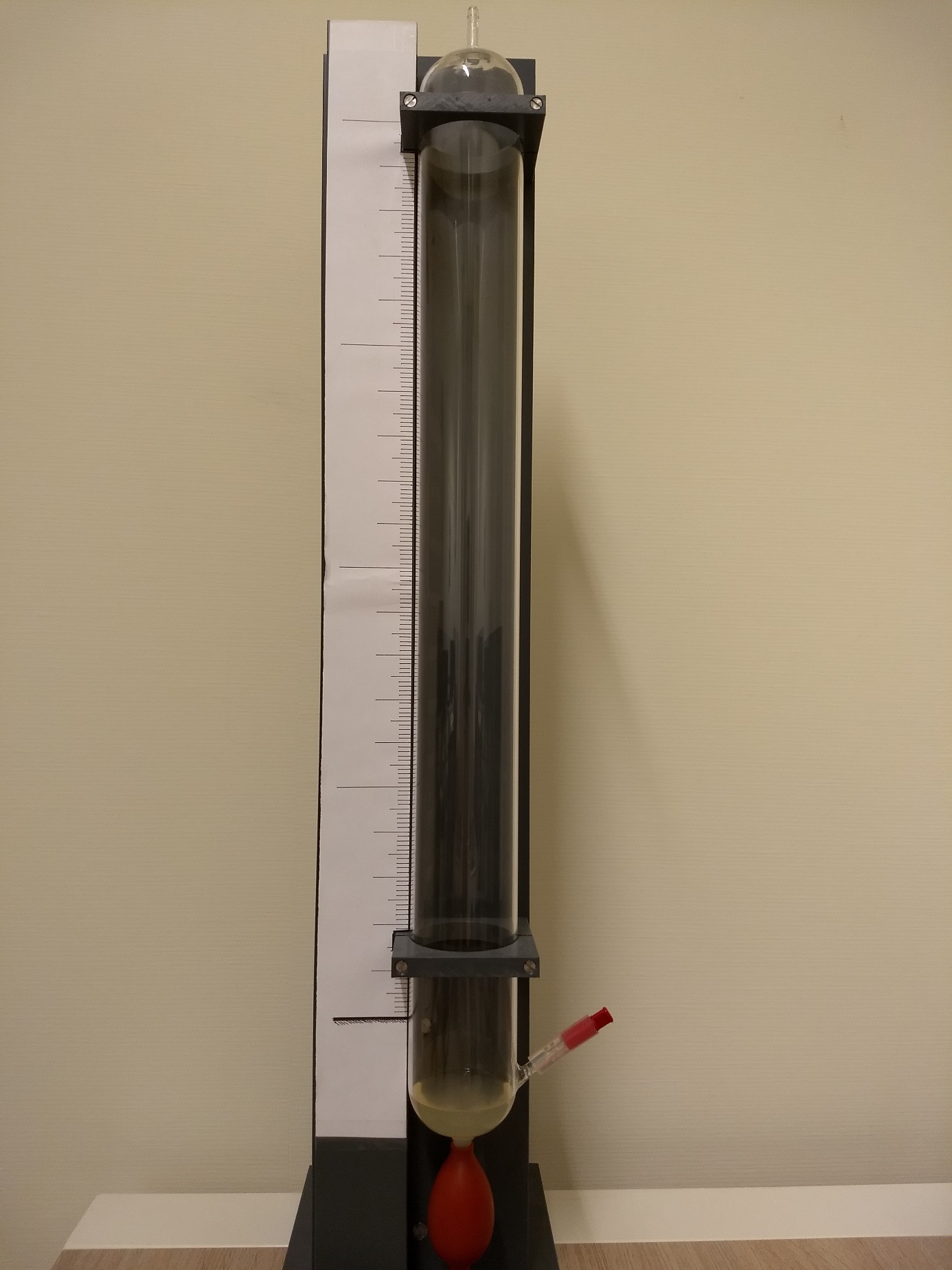
**

**Figure E3**
